# Metabolomic Analysis and Phenylpropanoid Biosynthesis in Hairy Root Culture of Tartary Buckwheat Cultivars

**DOI:** 10.1371/journal.pone.0065349

**Published:** 2013-06-14

**Authors:** Aye Aye Thwe, Jae Kwang Kim, Xiaohua Li, Yeon Bok Kim, Md Romij Uddin, Sun Ju Kim, Tatsuro Suzuki, Nam Il Park, Sang Un Park

**Affiliations:** 1 Department of Crop Science, Chungnam National University, Daejeon, Republic of Korea; 2 National Academy of Agricultural Science, Rural Development Administration, Suwon, Republic of Korea; 3 Department of Bio Environmental Chemistry, Chungnam National University, Daejeon, Republic of Korea; 4 National Agricultural Research Center for Hokkaido Region, Hokkaido, Japan; 5 Wildlife Genetic Resources Center, National Institute of Biological Resources, Incheon, Republic of Korea; Imperial College London, United Kingdom

## Abstract

Buckwheat, *Fagopyrum tataricum* Gaertn., is an important medicinal plant, which contains several phenolic compounds, including one of the highest content of rutin, a phenolic compound with anti-inflammatory properties. An experiment was conducted to investigate the level of expression of various genes in the phenylpropanoid biosynthetic pathway to analyze *in vitro* production of anthocyanin and phenolic compounds from hairy root cultures derived from 2 cultivars of tartary buckwheat (Hokkai T8 and T10). A total of 47 metabolites were identified by gas chromatography–time-of-flight mass spectrometry (GC-TOFMS) and subjected to principal component analysis (PCA) in order to fully distinguish between Hokkai T8 and T10 hairy roots. The expression levels of phenylpropanoid biosynthetic pathway genes, through qRT-PCR, showed higher expression for almost all the genes in T10 than T8 hairy root except for *FtF3’H-2* and *FtFLS-2*. Rutin, quercetin, gallic acid, caffeic acid, ferulic acid, 4-hydroxybenzoic acid, and 2 anthocyanin compounds were identified in Hokkai T8 and T10 hairy roots. The concentration of rutin and anthocyanin in Hokkai T10 hairy roots of tartary buckwheat was several-fold higher compared with that obtained from Hokkai T8 hairy root. This study provides useful information on the molecular and physiological dynamic processes that are correlated with phenylpropanoid biosynthetic gene expression and phenolic compound content in *F. tataricum* species.

## Introduction

Phenylpropanoids are a diverse group of compounds derived from the carbon skeleton of phenylalanine, which are involved in plant defense, structural support, and survival [1]. The phenylpropanoid pathway serves as a rich source of metabolites in plants, being required for the biosynthesis of lignin and serving as a starting point for the production of many other important compounds such as flavonoids, coumarins, and lignans [2].

Buckwheat (*Fagopyrum* sp.), which is an important nutrient-rich pseudocereal crop, belongs to the family Polygonaceae. The crop can be used as grain or as a green vegetable and has a nutraceutical value rich in carbohydrates. There are 2 buckwheat (*Fagopyrum*) species, common buckwheat (*Fagopyrum esculentum* Moench.) and tartary buckwheat (*Fagopyrum tataricum* Gaertn.). A tartary buckwheat cultivar Hokkai T8 was bred by pureline selection from a Russian cultivar, Rotundatum, whereas Hokkai T10 was obtained through ethyl methane sulfonate (EMS) mutagenesis of Hokkai T8. Anthocyanins identified in the sprouts of Hokkai T10 include cyanidin 3-*O-*glucoside (C3gl) and cyanidin 3-*O*-rutinoside (C3r) [3].

Both species of buckwheat, common and tartary, are major sources of rutin. In fact, rutin is found in high quantities in buckwheat and cannot be found in other grains such as wheat, rice, or corn. Thus, buckwheat is considered to be a major dietary source of rutin. Interestingly, seeds of tartary buckwheat contain 40–50 times higher rutin than those of common buckwheat [4]. Rutin has desirable physiological and biological properties such as anti-oxidant, anti-inflammatory, anti-hypertensive, vasoconstrictive, spasmolytic properties, as well as positive inotropic effects [5], [6]. It also provides protection against gastric lesions, improves sight and hearing, protects against UV light [7], lowers plasma cholesterol [8], protects from oxidative stress [9], causes muscle hypertrophy [8], and suppresses gallstone formation and cholesterol levels [5]. Tartary buckwheat has been reported to exhibit various pharmacological and biological activities, including anticancer [10], antidiabetic [11], and antioxidant activities [12]. It is well known that the organs of buckwheat (leaf, stem, and inflorescence) contain several phenolic compounds. Hairy root cultures of many plant species have been widely studied for the production of secondary metabolites useful as pharmaceuticals, cosmetics, and food additives [13], [14], [15]. Biotechnological production of rutin by hairy root culture of common buckwheat has also been reported [16].

The phenolic acids are derived from central or primary metabolic processes in plants. Primary metabolite profiling allows for classification of samples with diverse biological status, origin, or quality using chemometrics such as principal component analysis (PCA). The primary metabolite profile is closely related to the organism’s phenotype and includes important nutritional characteristics [17]. The primary application of metabolomics in plants includes screening mutant collections [18], quality control and quality assessment of food and crop products [19], and development of traditional medicines [20].

**Figure 1:**
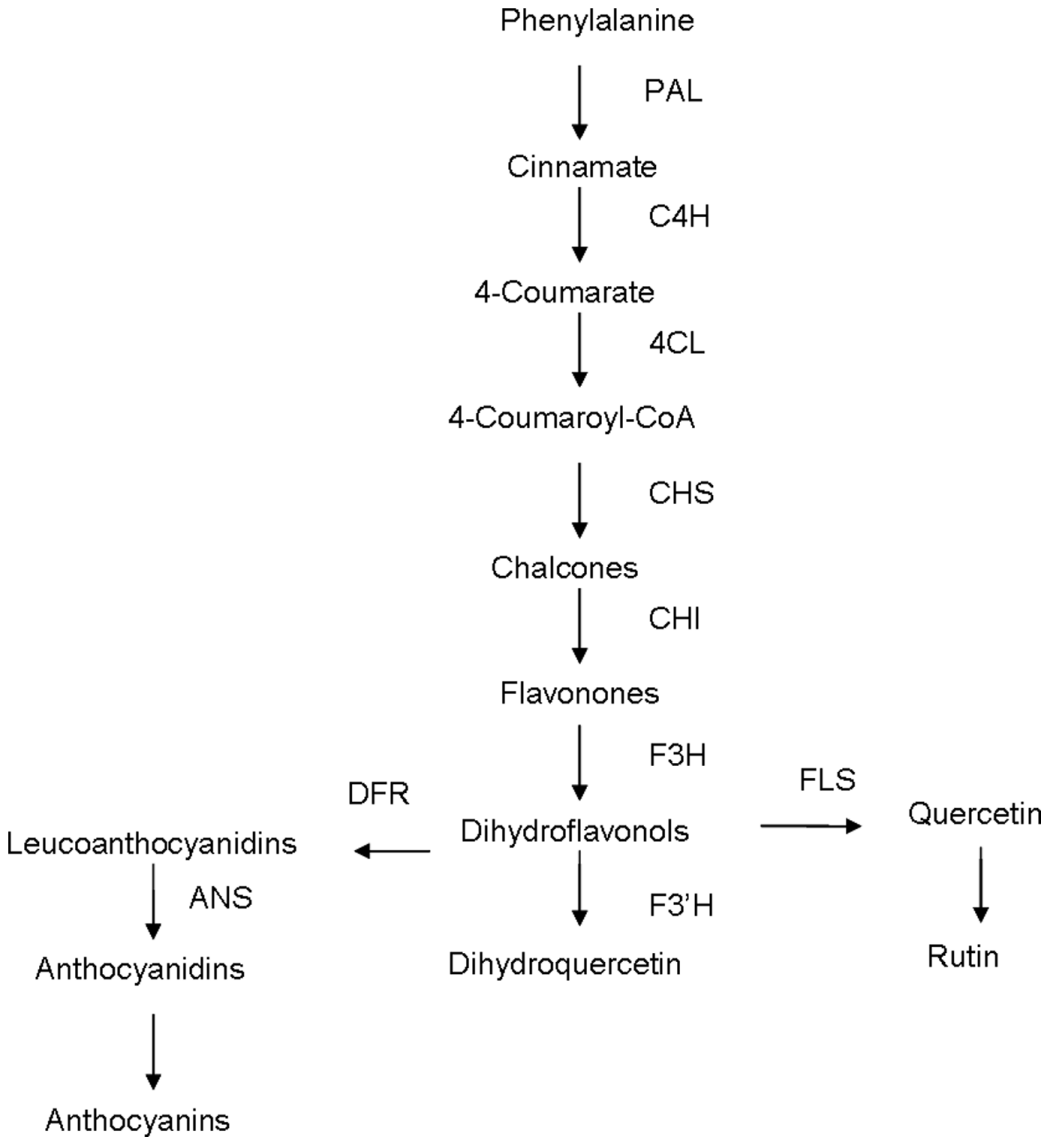
Phenylpropanoid biosynthesis pathway in *F. tataricum*. *PAL*, phenylalanine ammonialyase; *C4H*, cinnamate 4-hydroxylase; *4CL*, 4-coumaroyl CoA ligase; *CHS*, chalcone synthase; *CHI*, chalcone isomerase, *F3H*, flavone 3-hydroxylase, *DFR*, dihydroflavonol 4-reductase; *FLS*, flavonol synthase; *F3′H*, flavonoid 3′-hydroxylase; *ANS*, anthocyanidin synthase.

Flavonoids are a class of secondary metabolites in plants that are involved in many important functions. They constitute a relatively diverse group of aromatic compounds that are derived from phenylalanine and malonyl-coenzyme [21]. Phenylalanine ammonia lyase (PAL) catalyzes the conversion of phenylalanine to cinnamate. Following this, the cinnamate 4-hydroxylase (C4H) catalyzes the hydroxylation of trans-cinnamic acid to *p*-coumaric acid [22]. A simple illustration for flavonoid biosynthesis pathway in *F. tataricum* is presented in Figure 1.

Hairy root cultures, established by transformation with *A. rhizogenes*, are attractive for the production of secondary metabolites. As such, they are genetically and biochemically stable, show rapid growth rates, and have the ability to synthesize useful natural compounds at levels comparable to those produced by wild-type roots [23], [24]. The *rol* (root loci) genes belong to the T-DNA of *A. rhizogenes* Ri (root-inducing) plasmid [25], [26], [27]. Thus, in natural *A. rhizogenes* plant infections, *rol* genes, together with other genes of the T-DNA, are transferred to the plant genome. This transfer produces hairy root disease, a proliferation of roots at the site of infection. The *rol* genes have been related to rhizogenesis and are produced in the infected tissues [28], [29]. *rol* A, B, C, and D genes have been identified as the main determinants of hairy root disease which affects dicotyledonous plants and is caused by the soil bacterium, *A. rhizogenes* [30]. *A. rhizogenes* infects the host tissue through the wounds of many plant species and the infections are characterized by production of numerous root hairs [31], [32]. Therefore, hairy root cultures can be useful tools in genetic studies, as well as in the production of plant secondary metabolites.

In this study, we describe *Agrobacterium rhizogenes*–mediated hairy root induction of the biosynthesis and accumulation of phenolic compounds in different tartary buckwheat hairy roots (Hokkai T8 and T10) along with the expression of phenylpropanoid biosynthesis pathway genes through quantitative real time PCR. In addition, hydrophilic metabolic profiling (including phenolics) in tartary buckwheat using gas chromatography–time-of-flight mass spectrometry (GC-TOFMS) was applied to determine the phenotypic variation and analyze relationships between their contents.

## Materials and Methods

### Seed Disinfection and Germination

The seeds of *F. tataricum* (Hokkai T8 and T10) were procured from the National Agricultural Research Center (Hakkaido, Japan). For hairy root induction, gene expression and chemical analysis of phenylpropanoid biosynthetic pathway genes, the experiment was started by disinfection of the seeds in 70% (v/v) ethanol for 30 s and 4% (v/v) sodium hypochlorite solution (NaClO) for 10∼15 min. Seeds were then rinsed thoroughly in sterilized distilled water, blotted dry on sterile tissue paper and incubated in 25 ml of hormone-free half-strength MS [33] basal medium solidified with 0.8% (w/v) plant agar in Petri dishes. The pH of the medium was adjusted to 5.8 prior to adding plant agar, and sterilized by autoclaving at 121°C for 20 min. The seeds were germinated for three days under standard cool white fluorescent tubes with a flux rate of 35 µmol s^−1^ m^−2^ and transferred to a Magenta box containing 50 ml of the same basal medium and grown under light and dark condition (16/8 h) until use.

### Chemicals and Standards

The standards (rutin, quercetin, gallic acid, caffeic acid, ferulic acid, 4-hydroxybenzoic acid) were purchased from Extrasynthese (Genay, France); Cyanidin-3-*O*-glucoside was purchased from Fujicco Co., Ltd. (Kobe, Japan); Cyanidin-3-*O*-rutinoside from ChromaDex, Inc. (Irvine, CA); and formic acid and acetonitrile were obtained from commercial sources.

### Hairy Root Induction by *A. rhizogenes*

A wild type *A. rhizogenes* (R1000 strain) was provided by Dr. Victor Loyola-Vargas and Dr. Felipe Vázquez-Flota (Centro de Investigación Cientifica de Yucatán, México).The bacterial cells were cultured in the flask containing 30 ml of LB liquid medium, at 28°C, in a rotary shaker until mid-log phase (A_600_ = 0.5). Then, the cell broth was centrifuged at 4°C with 3000 rpm for 10 min to collect the incubated cells. Cell pellets were then resuspended in half-strength MS liquid medium for plant inoculation. Hypocotyl parts of in vitro grown 10 days old *F. tataricum* seedlings were aseptically cut into small sections of (∼8 mm), infected with the previously cultured bacterial inoculum for 10∼15 min. Thereafter, they were blotted dry on sterilized tissue paper, co-cultured on hormone-free agar solidified half MS medium and incubated under dark conditions for two days. The co-cultured explants tissues were then washed thoroughly with sterilized distilled water and transferred to a hormone-free half MS medium supplemented with 500 mg/l cefotaxime (LPS SOLUTION, LPSS, Daejeon, Korea), Fresh growing hairy roots were obtained after 3∼4 times passage into the fresh the medium. These hairy roots were transferred to the flask containing 30 ml of half MS liquid medium, and maintained at 25°C on a shaker (100 rpm) under 24 h dark condition. Hairy roots were harvested after 30 days of culture on shaker for extraction of genomic DNA, total RNA, and analysis of anthocyanin and phenolic compounds. The harvested samples were carefully handled and immediately stored in −80°C for further analysis.

### Genomic DNA Extraction and PCR Analysis

To confirm the insertion of *rol* genes in *A. rhizogenes* mediated transgenic tartary buckwheat hairy roots and wild type (seedling) roots, plant genomic DNA was extracted by using QIAGEN DNeasy Plant Mini Kit. PCR amplification program included 95°C for 2 min, 30 cycles of 95°C for 30 s, annealing temperature (55–50°C) for 45 s, 72°C for 1 min and a final extension of 10 min at 72°C. After the amplification, 10 µl of amplified PCR products were mixed with loading dye and electrophoresed on 1% agarose gels prepared in 0.5× TBE (Tris/Borate/EDTA) buffer. Gels were analyzed using gel documentation system for the determination of respective *rol* genes (*rol* A, B, C, and D) fragment sizes. The primer sequences information was listed in Table S1.

### Total RNA Extraction and cDNA Synthesis

Total RNA was isolated from *F. tataricum* wild and transgenic hairy roots by using the RNeasy Plant Mini Kit (Qiagen; Valencia, CA, USA). The RNA integrity was checked by 1.2% ethidium bromide stained RNA gel and through the absorbance spectrum at 260∶280 nm wavelength by NanoVue Plus Spectrophotometer (GE Healthcare Bio-Science Crop, USA). The cDNA was synthesized from 1 µg of DNA free total RNA and reverse-transcribed using reverse transcriptase with oligo (dT)_20_ primer (Toyobo, Japan). The resulting cDNA products were used as templates for real time-PCR analysis.

### Expression Analysis of Phenylpropanoid Biosynthetic Genes by qRT-PCR

Quantitative real-time PCR was performed for transcriptional level analysis of phenylpropanoid biosynthesis genes from *F. tataricum* hairy roots using a BIO-RAD CFX96 Real-time PCR system (Bio-Rad Laboratories, Hercules, CA). The gene-specific primer sets were designed as previous information described by [34]. Real-time PCR was carried out in a 20 µl reaction volume including 0.5 µl of each primer, 5 µl of template cDNA and 10 µl of SYBR Green (Toyobo) using the following conditions; 95°C for 3 min, followed by 40 cycles of 95°C for 10 s, annealing for 10 s at 55°C, and elongation for 30 s at 72°C. The histone H3 gene was using as reference gene [35]. Fluorescent intensity data were acquired during the extension step. The transcript levels were checked using a standard curve. Identical PCR conditions were used for all targets. The significant differences between cultivars were evaluated from three replicates of each sample.

### GC-TOFMS Analysis of Polar Metabolites

Hairy roots of *F. tataricum* were dried using the freeze-dryer at −80°C for at least 48 h. After drying, the samples were ground into a fine powder using a mortar and pestle. Polar metabolite extraction was performed as described previously [36]. A total of 20 mg of ground sample was extracted with 1 ml of a mixed solvent of methanol/water/chloroform (2.5∶1:1 by vol.). Ribitol solution (120 µl, 0.2 mg/ml) was added as an internal standard (IS). Extraction was performed at 37°C with a mixing frequency of 1200 rpm for 30 min, using a thermomixer compact (Eppendorf AG, Germany). The solutions were then centrifuged at 16,000×*g* for 3 min. The polar phase (0.8 ml) was transferred into a new tube, and 0.4 ml of water was added before centrifugation in order to separate the nonpolar phase. The mixed contents of the tube were centrifuged at 16,000×*g* for 3 min. The methanol/water phase containing hydrophilic metabolites was dried in a centrifugal concentrator (CVE-2000, Eyela, Japan) for 2 h, followed by a drying process in a freeze dryer for 16 h. Methoxime (MO)-derivatization was performed by adding 160 µl of methoxyamine hydrochloride (20 mg/ml) in pyridine and shaking at 30°C for 90 min. Trimethylsilyl (TMS) etherification was performed by adding 160 µl of MSTFA at 37°C for 30 min. Thus, hydrophilic metabolites were derivatized by silylation and oximation reactions: TMS from hydroxyl and primary amine groups and methoximation of carbonyl groups. GC-TOFMS was performed using an Agilent 7890A gas chromatograph (Agilent, Atlanta, GA, USA) coupled to a Pegasus HT TOF mass spectrometer (LECO, St. Joseph, MI). The derivatized sample (1 µL) was separated on a 30-m×0.25-mm I.D. fused-silica capillary column coated with 0.25-µm CP-SIL 8 CB low bleed (Varian Inc., Palo Alto, CA, USA). The split ratio was set at 1∶25. The injector temperature was 230°C. The helium gas flow rate through the column was 1.0 mL/min. The temperature program was as follows: Initial temperature 80°C for 2 min, followed by an increase to 320°C at 15°C/min, and a 10 min hold at 320°C. The transfer line and ion-source temperatures were 250 and 200°C, respectively. The scanned mass range was 85–600 *m/z*, and the detector voltage was set at 1700 V.

### Phenolic Compound Extraction and Quantification by HPLC

The freeze-dried hairy roots were ground into a fine powder and 100 mg of the samples were extracted with 3 ml of 100% methanol at 60°C for 1 h in sonicator. The samples were vortexed for every 20 minutes during extraction. After that, the sample mixtures were centrifuged at 4°C and 14,000 rpm for 10 minutes to get the extracted solution. The solution was then filtered through 0.45 µm PTFE syringe filter (Advantec DISMIC-13HP,

Toyo Roshi Kaisha, Ltd., Tokyo, Japan). After extraction, HPLC quantification of phenolic compounds was performed with a Futecs model NS-4000 HPLC apparatus (Daejeon, Korea) and followed according to the protocol previously described [34]. In detail, the mobile phase was a gradient consisted of a mixture of (A) MeOH:water:acetic acid (5∶92.5∶2.5, v/v/v) and (B) MeOH:water:acetic acid (95∶2.5∶2.5, v/v/v). The initial mobile phase composition was 0% solvent B, followed by a linear gradient from 0 to 80% of solvent B over 48 min, and then holding at 0% solvent B for an additional 10 min. The column was maintained at 30°C, the flow rate was 1.0 mL/min, the injection volume was 20 µl. The eluted components were monitored at 280 nm and performed using a C18 column (250 mm*4.6 mm, 5 µm; RStech, Daejeon, Korea). All the phenolic compounds were calculated by comparing the HPLC peak area with that of authentic standards following the procedures described previously [34].

### Anthocyanins Quantification by HPLC

For anthocyanin analysis, 100 mg of ground samples were placed in 2 ml eppendorf tubes. The solvent, water:formic acid 95∶5 (v/v),was prepared under dark conditions and ∼2 ml of this solvent was added to each samples for extraction. The sample tubes were vortexed for 5 minutes and incubated in sonicator for 20 minutes. Then the samples were centrifuged at 10,000 rpm for 15 min at 4°C, the supernatant was filtered using 0.45-µm hydrophilic syringe filter and injected into brown vials for analysis. The anthocyanin filtrate was analyzed using PerkinElmer flexar HPLC (PerkinElmer Inc., shelton, CT) equipped with a Synergi 4 μ POLAR-RP 80A column (250 mm×4.6 mm, i.d., particle size 4 µm; Phenomenex, Torrance, CA) equipped with a Security Guard Cartridges Kit AQ C18 column (4 mm×3 mm, i.d.). The mobile phase consisted of a mixture of (A) water/formic acid (95∶5, v/v) and (B) acetonitrile/formic acid (95∶5, v/v). The gradient program was set as: 0−8 min, 5−10% solvent B; 8−13 min, 10−13% solvent B; 13−15 min, 13% solvent B; 15−18 min, 13−15% solvent B; 18−25 min, 15% solvent B; 5% solvent B at 25.1 min; and finally 5% solvent B constant for 10 min (total 35 min). Detection was performed at 520 nm wavelength, and the column oven temperature was 40°C. The flow rate was set at 1.0 mL/min, and injection volume was 10 µL. The anthocyanin content was calculated by comparing the HPLC peak area with that of an authentic standard (cyanidin-3-*O*-glucoside and cyanidin-3-*O*-rutinoside).

### Statistical Analysis

The data for gene expression and phenolic compound contents were analyzed by using the computer software Statistical Analysis System (SAS version 9.2). Treatment means were compared by Duncan’s Multiple Range Test (DMRT). The relative quantification data acquired from GC-TOFMS was subjected to PCA (SIMCA-P version 12.0; Umetrics, Umeå, Sweden) to evaluate the relationships in terms of similarity or dissimilarity among groups of multivariate data. The PCA output consisted of score plots to visualize the contrast between different samples and loading plots to explain the cluster separation. The data file was scaled with unit variance scaling without any transformation.

## Results and Discussion

### Hairy Root Induction by *A. rhizogenes*

**Figure 2:**
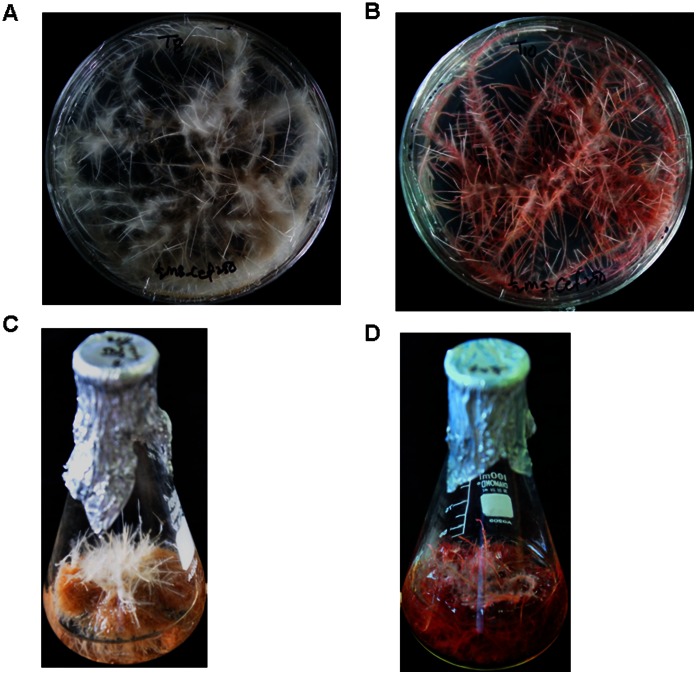
Growth of the hairy roots of F. tataricum after 30 days culture in hormone free MS liquid medium. Hokkai T8 (A, C) and Hokkai T10 (B, D).

Tartary buckwheat (*F. tataricum*) hairy roots were established by using hypocotyl parts inoculated with *A. rhizogenes* (R1000 strain). Numerous hairy roots initiated from wound sites of hypocotyl explants were subcultured on fresh agar-solidified medium repeatedly. These hairy roots grew very rapidly on the fresh solid medium (Figure 2A and 2B). The difference in the color of the hairy roots between Hokkai T8 and Hokkai T10 became more conspicuous within 2 months of culture in liquid medium. Hokkai T8 hairy roots have a pure white color, whereas Hokkai T10 hairy roots have a deep reddish-purple color (Figure 2C and 2D) because of their difference in anthocyanin accumulation patterns. To determine the insertion of *rol* genes, PCR was performed by using specific primer sets of *rol* A, B, C, and D genes (Table S1). The control (wild-type) root (lanes 1, 3, 5, 7) was negative for *rol* genes, whereas hairy roots (lane 2, 4, 6, 8) gave the expected bands for *rol* A (304 bp), B (797 bp), C (550 bp), and D (1035 bp) genes (data not shown). It was evident from these results that the hairy roots had *rol* gene inserts from the Ri plasmid.

### Expression of Phenylpropanoid Biosynthesis Genes in Hairy Roots of *F. tataricum*

**Figure 3:**
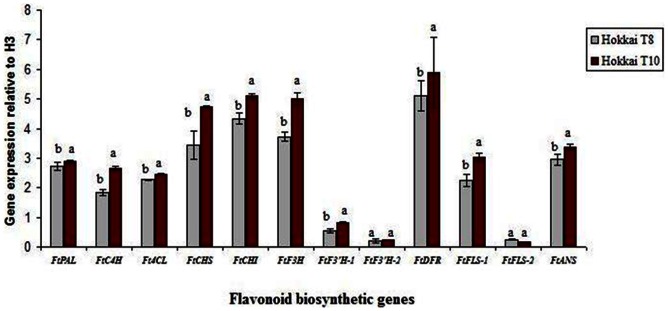
Expression levels of flavonoid biosynthesis genes in hairy roots of *F. tataricum.* The expression level of each gene is relative to that of the constitutively expressed histone H3 gene.

To investigate biosynthesis of phenylpropanoid genes in *F. tataricum*, the expression levels of biosynthesis genes in hairy roots of *F. tataricum* (Hokkai T8 and T10) were examined. The expression levels of *FtPAL*, *FtC4H*, *Ft4CL*, *FtCHS*, *FtCHI*, *FtF3H*, *FtF3′H-1*, *FtF3′H-2*, *FtDFR*, *FtFLS-1*, *FtFLS-2*, and *FtANS* are shown in Figure 3. Although the gene transcripts for all of these enzymes were expressed in hairy roots (T8 and T10) of *F. tataricum*, expression levels were upregulated in T10 than T8 hairy roots except in *FtF3’H-2* and *FtFLS-2*. In particular, expression levels of *FtPAL, FtC4H, Ft4CL, FtCHS, FtCHI, FtF3H and FtF3’H-1* in Hokkai T10 hairy roots were significantly higher than T8 hairy roots. Similarly, *FtDFR, FtFLS-1,* and *FtANS*, which are the key enzymes involved in anthocyanin biosynthetic pathway, were also significantly expressed in T10 than T8. Our results concurred with [37] who reported the expression of *FtANS* was significantly higher in ‘Hokkai T10’ than in ‘Hokkai T8’ roots. This finding shows that one of the anthocyanin biosynthetic gene, *FtANS,* is obviously responsive for anthocyanin pigment formation not only in normal roots but also in Agrobacterium mediated transformed hairy roots of the same cultivar, Hokkai T10. However, *FtF3′H-1*, *FtF3′H-2*, and *FtFLS-2*, which are isomers of *FtF3H* and *FtFLS*, showed at low levels compared with other genes in both hairy root types. In addition, *FtF3’H-2* and *FtFLS-2* genes showed no significant differences in expression between the two cultivars.

### Content of Phenolic Compounds in Tartary Buckwheat Hairy Roots

Rutin, quercetin, gallic acid, caffeic acid, ferulic acid, and 4-hydroxybenzoic acid were identified in hairy roots of tartary buckwheat (Table 1). Irrespective of cultivars, the main compounds observed in both cultivars were rutin and caffeic acid. With respect to cultivar, the concentration of rutin was the highest followed by caffeic acid among the analyzed compounds, and that of gallic acid was the lowest in T10 hairy root. In contrast, caffeic acid was the highest compound followed by rutin and the lowest was quercetin in T8 hairy root. Specifically, amount of rutin in T10 hairy root was 59.02 µg/mg and that in T8 was 0.25 µg/mg. This implies that rutin accumulates more than 200 fold in T10 compared with T8 hairy root.

**Table 1:** Content of phenolic compounds (µg/mg dry weight) in hairy root culture of tartary buckwheat cultivars Hokkai T8 and T10.

Phenolic compound	T8 hairy root	T10 hairy root
Rutin	0.252±0.009	59.015±2.621
Quercetin	0.003±0.001	0.428±0.001
Gallic acid	0.037±0.001	0.043±0.003
Caffeic acid	0.511±0.004	0.501±0.006
Ferulic acid	0.013±0.002	0.487±0.005
4-Hydroxybenzoic acid	0.037±0.003	0.16±0.007

Values represent the mean ± SD (n = 3).

Accumulation of phenolic compound in wild and hairy root of *F.tataricum* was compared [38]. They reported that phenolic content in hairy roots were several fold higher than wild type roots of same species. Furthermore, they observed epicatechin and rutin were the main compounds found in both types of root. According to their study, phenolic compounds accumulate more in *A. rhizogenes* transformed hairy root compared with wild type from mother plant. It has been reported that buckwheat is a rich source of rutin [39]. Moreover, rutin has been discovered to be the main compound in buckwheat [40]. In a recent study, except for caffeic acid, the amounts of quercetin, gallic acid, ferulic acid, and 4-hydroxybenzoic acid were higher in T10 when compared with the content in T8 hairy roots. The accumulation of 4-hydroxybenzoic acid in *A. rhizogenes*–induced hairy root cultures of *Daucus carota* was also reported [41]. Ferulic acid is contained in trace amounts in both hairy root types. This finding concurred with that of Tsuzuki and Yamamoto (1987) [42], according to which buckwheat contained a trace amount of ferulic acid. However, tomato hairy roots yielded ferulic acid as the major phenolic compound [43].

### Anthocyanin Content in Transgenic Hairy Roots

The content of anthocyanins in Hokkai T8 and Hokkai T10 hairy roots are presented in Table 2. Anthocyanin contents were detected in T10 but not in T8 hairy roots. In T10 hairy roots, cyanidin 3-*O*-glucoside content was found to be higher than cyanidin 3-*O*-rutinoside. This may be due to the greater level of anthocyanin pigment accumulation in T10 than T8. It has been noted that all the plant organs from Hokkai T10 cultivar contained more anthocyanin than T8 [37]. Detection of anthocyanin in Hokkai T10 hairy roots is related to higher gene expression of *FtF3H* and *FtANS* in the flavonoid biosynthesis pathway. In our results, we found that both these enzymes are critical for cyanidin 3-*O*-glucoside and cyanidin 3-*O*-rutinoside biosynthesis.

**Table 2:** Anthocyanin content (mg/g dry weight) in Hokkai T8 and Hokkai T10 in hairy roots.

Compound	T8 hairy root	T10 hairy root
Cyanidin 3-*O*-glucoside	ND	2.123±0.01
Cyanidin 3-*O*-rutinoside	ND	1.986±0.01

Values represent the mean ± SD (n = 3). ND = not detected.

### Metabolic Profile in Hairy Roots of *F. tataricum*

**Figure 4:**
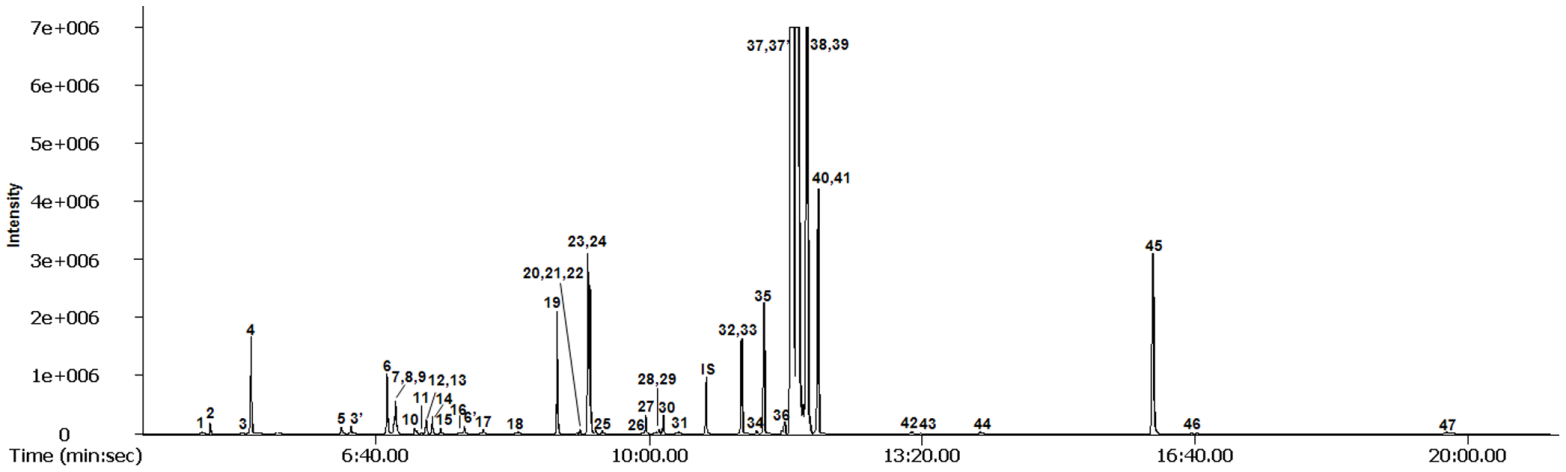
Selected izon chromatograms of metabolites extracted from tartary buckwheat (cv. T10) as MO/TMS derivatives separated on a 30 m×0.25-mm I.D. fused-silica capillary column coated with 0.25-µm CP-SIL 8 CB low bleed. Peak identification: 1, pyruvic acid; 2, lactic acid; 3, valine; 4, alanine; 5, glycolic acid; 3′, valine; 6, serine; 7, ethanolamine; 8, glycerol; 9, leucine; 10, isoleucine; 11, proline; 12, nicotinic acid; 13, glycine; 14, succinic acid; 15, glyceric acid; 16, fumaric acid; 7′, serine; 17, threonine; 18, β-alanine; 19, malic acid; 20, salicylic acid; 21, aspartic acid; 22, methionine; 23, pyroglutamic acid; 24, 4-aminobutyric acid; 25, threonic acid; 26, arginine; 27, glutamic acid; 28, phenylalanine; 29, *p*-hydroxybenzoic acid; 30, xylose; 31, asparagine; 32, vanillic acid; 33, glutamine; 34, shikimic acid; 35, citric acid; 36, quinic acid; 37, fructose; 37, fructose; 38, galactose; 39, glucose; 40, syringic acid; 41, mannose; 42, inositol; 43, ferulic acid; 44, tryptophan; 45, sucrose; 46, trehalose; 47, raffinose; IS, internal standard (ribitol).

**Figure 5:**
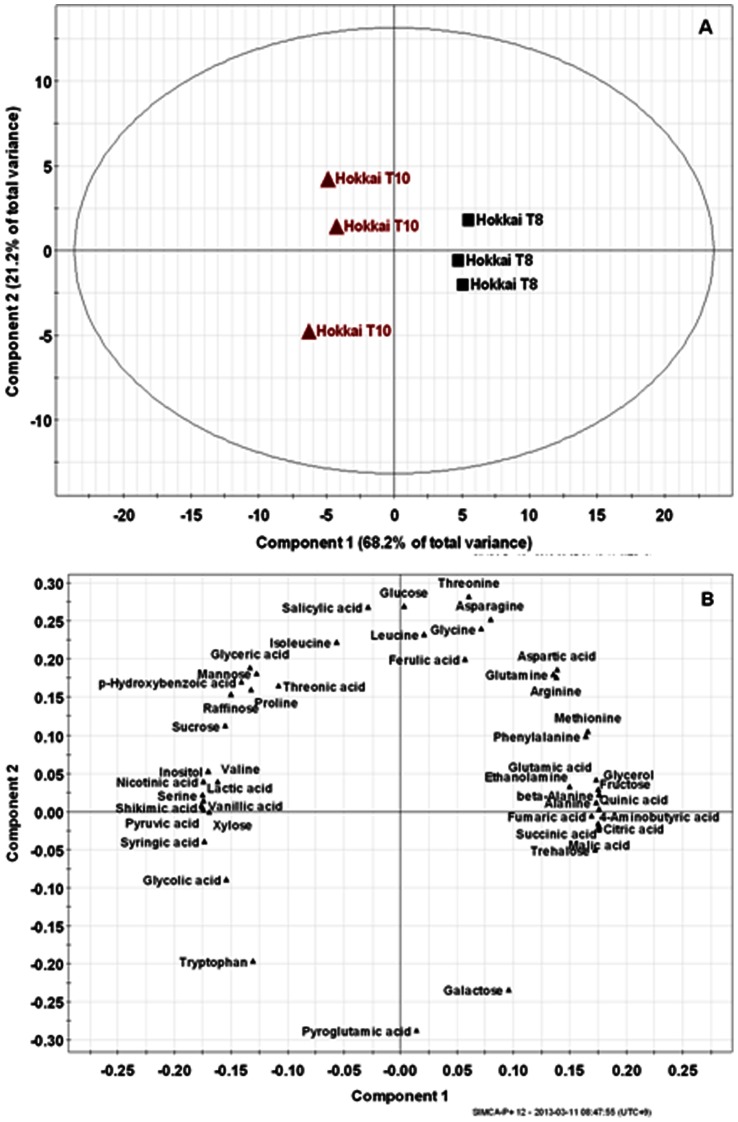
Scores (A) and loading (B) plots of principal components 1 and 2 of the PCA results obtained from metabolite data derived from tartary buckwheat cultivars.

The production of secondary metabolites is tightly associated with pathways of primary metabolism. Thus, we conducted comprehensive metabolic phenotyping of the primary metabolism in hairy roots of *F. tataricum* (Hokkai T8 and T10). In this study, low-molecular weight molecules from hairy roots were identified by GC-TOFMS. The main advantage of GC-TOFMS is that it enables spectra to be accumulated rapidly, thereby increasing the speed of GC-MS analyses, making them ideal for the analysis of complex mixtures. ChromaTOF software was used to assist with peak location. Peak identification was performed by comparison with reference compounds and the use of an in-house library. In addition, identification of several metabolites was performed using direct comparison of the sample mass chromatogram with those of commercially available standard compounds, which were obtained by a similar methoxime (MO)/trimethylsilyl (TMS) derivatization and GC-TOFMS analysis. In total, 47 metabolites, including 19 amino acids, 17 organic acids, 8 sugars, 2 sugar alcohols, and 1 amine were detected in *F. tataricum* (Figure 4). The corresponding retention times and their fragment patterns are illustrated in Table S2 and agree with the previous data [36], [44]. Among these metabolites, 5 phenolics (ferulic, *p*-hydroxybenzoic, salicylic, syringic, and vanillic acids) were identified in the samples. Quantification was performed using selected ions (Table S2). The quantitative calculations of all analytes were based on the peak area ratios relative to that of the IS. The data for the 47 metabolites were subjected to PCA to assess the overall experimental variation and to outline the differences in metabolite profiles between Hokkai T8 and T10 (Figure 5). PCA revealed that the 2 highest ranking principal components accounted for 89.4% of the total variance within the data set. The first principal component, accounting for 68.2% of total variance, resolved the measured metabolite profiles of T8 and T10 hairy roots (Figure 5A). To investigate the contributors to the principal components further, the metabolic loading in principal component 1 (PC1) was compared (Figure 5B). The significant metabolites for PC1 were shikimic, pyruvic and lactic acid. In the PC1, the corresponding loading was positive for tricarboxylic (TCA) cycle intermediates such as citric, fumaric and succinic acid, while negative for all phenolic acids, excluding ferulic acid as well as shikimic, pyruvic and lactic acid. These results revealed correlations between metabolites that participate in closely related pathways and demonstrated the robustness of the present experimental system.

The building blocks for secondary metabolites are derived from primary metabolism. The loading plot of PC1 indicated that a higher amount of phenolic, shikimic, pyruvic and lactic acid was present in the T10 hairy roots and TCA cycle intermediates levels were higher in the T8 hairy roots. Among the phenylpropanoids, the subgroup of hydroxycinnamic acids is very common in plants, e.g. ferulic, *p*-coumaric and *p*-hydroxybenzoic acid, usually occurring as esters of sugars, organic acids or amino acids. Shikimic acid, one of the important building blocks employed in the biosynthesis of phenylpropanoids, is produced from a combination of phosphoenolpyruvate, a glycolytic pathway intermediate, and erythrose 4-phosphate from the pentose phosphate pathway. As previously reported [45], the GC-TOFMS-based metabolic profiling approach could be used as an alternative method to identify metabolic links in complex biological systems.

### Conclusion

In this study, we described an efficient *A. rhizogenes*–mediated transformation protocol for the establishment of tartary buckwheat hairy root cultures (Hokkai T8 and T10). Specifically, we examined the levels of gene expression involved in the phenylpropanoid biosynthetic pathway and analyzed phenolic compound production and anthocyanin accumulation from transformed roots of *F. tataricum* (T8 and T10) using *A. rhizogenes* R1000. Moreover, GC-TOFMS was used to identify differences in metabolite profiles between transformed T8 and T10 hairy roots. PC 1 of the PCA indicated that a higher amount of phenolic, shikimic, pyruvic and lactic acid was present in the T10 hairy roots and TCA cycle intermediates levels were higher in the T8 hairy roots. Phenolic acids are produced from the shikimate pathway as secondary metabolites. This suggests that metabolomics could assist in dissecting mechanisms regulating the conversion of primary to secondary metabolism in plants. Hairy root induction by *A. rhizogenes* mediated transformation could be proved as an alternative approach for the production of phenolic compounds from different kinds of medicinal plants including buckwheat.

## Supporting Information

Table S1 PCR primer sets for amplification of *rol* genes.
(DOC)

Table S2 Chromatographic and spectrometric data of the 47 identified compounds analyzed by GC-TOFMS.
(DOC)

## References

[1] Vogt T (2010) Phenylpropanoid Biosynthesis. Molecular Plant 3: 2–20. 20035037 10.1093/mp/ssp106

[2] Fraser CM, Chapple C (2011) The phenylpropanoid pathway in Arabidopsis. The. Arabidopsis Book, e0152. 10.1199/tab.0152 PMC3268504 22303276

[3] Suzuki T, Watanabe M, Iki M, Aoyagi Y, Kim SJ, et al (2009) Time-course study and effects of drying method on concentrations of γ-aminobutyric acid, flavonoids, anthocyanin, and 2′′hydroxynicotianamine in leaves of buckwheats. J Agric Food Chem 57: 259–264. 19090759 10.1021/jf802731d

[4] Gupta N, Sharma SK, Rana JC, Chauhan RS (2011) Expression of flavonoid biosynthesisgenes vis-à-vis rutin content variation in different growth stages of Fagopyrum species. J Plant Physiol 168: 2117–2123. 21872967 10.1016/j.jplph.2011.06.018

[5] Kuntić V, Filipović I, Vujić Z (2011) Effects of rutin and hesperidin and their Al(III) and Cu(II) complexes on in vitro plasma coagulation assays. Molecules 16: 1378–1388. 21301410 10.3390/molecules16021378 PMC6259837

[6] Landberg R, Sun Q, Rimm EB, Cassidy A, Scalbert A, et al (2011) Selected dietary flavonoids are associated with markers of inflammation and endothelial dysfunction in U.S. women. The Journal of Nutrition 141: 618–625. 21325476 10.3945/jn.110.133843 PMC3057665

[7] Gaberščik A, Vončina M, Trošt T, Germ M, Björn LO (2002) Growth and productionof buckwheat (*Fagopyrum esculentum*) treated with reduced, ambient, and enhanced UV-B radiation. J Photochem Photobiol B:Biol 66: 30–36. 10.1016/s1011-1344(01)00272-x 11849980

[8] Kayashita J, Shimaoka I, Nakajoh M, Yamazaki M, Kato N (1997) Consumption of buckwheat protein lowers plasma cholesterol and raises fecal neutral sterols in cholesterol-fed rats because of its low digestibility. The Journal of Nutrition 127: 1395–1400. 9202097 10.1093/jn/127.7.1395

[9] Gong G, Qin Y, Huang W, Zhou S, Yang X, et al (2010) Rutin inhibits hydrogen peroxide-induced apoptosis through regulating reactive oxygen species mediated mitochondrial dysfunction pathway in human umbilical vein endothelial cells. European J Pharmacol 628: 27–35. 19931526 10.1016/j.ejphar.2009.11.028

[10] Guo X, Zhu K, Zhang H, Yao H (2007) Purification and characterization of the anti-tumor protein from Chinese tartary buckwheat (*Fagopyrum tataricum* Gaertn.) water-soluble extracts. J Agric Food Chem 55: 6958–6961. 17661488 10.1021/jf071032+

[11] Yao Y, Shan F, Bian J, Chen F, Wang M, et al (2008) D-chiro-Inositol-enriched tartary buckwheat bran extract lowers the blood glucose level in KK-Ay mice. J Agric Food Chem 56: 10027–10031. 18921966 10.1021/jf801879m

[12] Liu CL, Chen YS, Yang JH, Chiang BHA (2008) Antioxidant activity of tartary (*Fagopyrum tataricum* Gaertn.) and common (*Fagopyrum esculentum* Moench) buckwheat sprouts. J Agric Food Chem 56: 173–178. 18072736 10.1021/jf072347s

[13] Christey MC, Braun RH (2005) Production of hairy root cultures and transgenic plants by *Agrobacterium rhizogenes*-mediated transformation. Methods in Mol Biol 286: 47–60. 15310912 10.1385/1-59259-827-7:047

[14] Georgiev MI, Pavlov AI, Bley T (2007) Hairy root type plant in vitro systems as sourcesof bioactive substances. Appl Microbiol Biotechnol 74: 1175–1185. 17294182 10.1007/s00253-007-0856-5

[15] Srivastava S, Srivastava AK (2007) Hairy root culture for mass-production of high value secondary metabolites. Crit Rev Biotechnol 27: 29–43. 17364688 10.1080/07388550601173918

[16] Lee SY, Cho SI, Kim YK, Lee SY, Park MH, et al (2007) Growth and rutin production in hairy root cultures of buckwheat (*Fagopyrum esculentum* M.). Prep Biochem Biotechnol 37: 239–246. 17516253 10.1080/10826060701386729

[17] Kok EJ, Keijer J, Kleter GA, Kuiper HA (2008) Comparative safety assessment of plant-derived foods. Regulatory Toxicol Pharmacol 50: 98–113. 10.1016/j.yrtph.2007.09.007 17983697

[18] Messerli G, Partovi NV, Trevisan M, Kolbe A, Schauer N, et al (2007) Rapid classification of phenotypic mutants of Arabidopsis via metabolite fingerprinting. Plant Physiol 143: 1484–1492. 17277092 10.1104/pp.106.090795 PMC1851843

[19] Pongsuwan W, Fukusaki E, Bamba T, et al (2007) Prediction of Japanese green tea ranking by gas chromatography/mass spectrometry-based hydrophilic metabolite fingerprinting. J Agric Food Chem 55: 231–236. 17227047 10.1021/jf062330u

[20] Tarachiwin L, Masako O, Fukusaki E (2008) Quality evaluation and prediction of Citrullus lanatus by 1H NMR-based metabolomics and multivariate analysis. J Agric Food Chem 56: 5827–5835. 18588311 10.1021/jf800418u

[21] Winkel-Shirley B (2001) Flavonoid biosynthesis. a colorful model for genetics, biochemistry, cell biology, and biotechnology. Plant Physiol 126: 485–493. 11402179 10.1104/pp.126.2.485 PMC1540115

[22] Russell DW (1971) The metabolism of aromatic compounds in higher plants. J Biol Chem 246: 3870–3878. 4397825

[23] Guillon SH, Tremouillaux-Guiller J, Pati PK, Rideau M, Gantet P (2006a) Hairy root research: recent scenario and exciting prospects. Curr Opin Plant Biol 9: 341–346. 16616871 10.1016/j.pbi.2006.03.008

[24] Guillon SH, Tremouillaux-Guiller J, Pati PK, Rideau M, Gantet P (2006b) Har- nessing the potential of hairy roots: dawn of a new era. Trends Biotechnol 24: 403–409. 16870285 10.1016/j.tibtech.2006.07.002

[25] Filetici P, Spanò L, Costantino P (1987) Conserved regions in the T-DNA of different *Agrobacterium rhizogenes* root-inducing plasmids. Plant Mol Biol 9: 19–26. 24276794 10.1007/BF00017983

[26] Moriguchi K, Maeda Y, Satou M, et al (2001) The complete nucleotide sequence of a plant root-inducing (Ri) plasmid indicates its chimeric structure and evolutionary relationship between tumor-inducing (Ti) and symbiotic (Sym) plasmids in rhizobiaceae. J Mol Biol 307: 771–784. 11273700 10.1006/jmbi.2001.4488

[27] Petit A, David C, Dahl G, et al (1983) Further extension of the opine concept: plasmidsin *Agrobacterium rhizogenes* co-operate for opine degradation. Mol Genet Genomics 190: 204–214.

[28] Christey MC (2001) Use of ri-mediated transformation for production of transgenic plants. In Vitro Cell Dev Biol - Plant 37: 687–700.

[29] Tepfer D (1984) Transformation of several species of higher plants by *Agrobacterium rhizogenes*: Sexual transmission of the transformed genotype and phenotype. Cell 37: 959–967. 6744417 10.1016/0092-8674(84)90430-6

[30] Bettini P, Michelotti S, Bindi D, et al (2003) Pleiotropic effect of the insertion of the *Agrobacterium rhizogenes* rolD gene in tomato (*Lycopersicon esculentum* Mill.). Theoretical and Applied Genetics 107: 831–836. 12830385 10.1007/s00122-003-1322-0

[31] Giri A, Narusu ML (2000) Transgenic hairy roots: recent trends and applications. Biotechnol Adv 18: 1–22. 14538116 10.1016/s0734-9750(99)00016-6

[32] Hamill JD, Parr AJ, Rhodes MJC, Robins RJ, Walton NJ (1987) New routes to plant secondary products. Nat Biotechnol 5: 800–804.

[33] Murashige T, Skoog F (1962) A revised medium for rapid growth and bioassays with tobacco tissue culture. Physiol. Plant 15: 473–497.

[34] Li X, Park NI, Xu H, et al (2010) Differential expression of flavonoid biosynthesis genes and accumulation of phenolic compounds in common buckwheat (*Fagopyrum esculentum*). J Agric Food Chem 58: 12176–12181. 21062042 10.1021/jf103310g

[35] Timotijevic GS, Milisavljevic MD, Radovic SR, Konstantinovic MM, Maksimovic VR (2010) Ubiquitous aspartic proteinase as an actor in the stress response in buckwheat. J Plant Physiol 167: 61–68. 19643510 10.1016/j.jplph.2009.06.017

[36] Kim JK, Park SY, Lee SM, et al (2013) Unintended polar metabolite profiling of carotenoid biofortified transgenic rice reveals substantial equivalence to its non transgenic counterpart. Plant Biotechnology Reports 7: 121–128.

[37] Park NI, Li X, Suzuki T, et al (2011) Differential expression of anthocyanin biosyntheticgenes and anthocyanin accumulation in tartary buckwheat cultivars ‘Hokkai T8’ and ‘Hokkai T10’. Agric Food Chem 59: 2356–2361. 10.1021/jf200020b 21366292

[38] Kim YK, Li X, Xu H, Park NI, Uddin MR, et al (2009) Production of phenolic compounds in hairy root culture of tartary buckwheat (*Fagopyrum tataricum* Gaertn). J Crop Sci Biotech 12(1): 53–58.

[39] Lachman J, Orsak M, Pivec V, Faustusova E (2000) Content of rutin in selected plant sources. Sci Agric Boh 31: 89–99.

[40] Hagels H, Wagenbreth D, Schilcher H (1995) Phenolic compounds of buckwheat herb and influence of plant and agricultural factors (*Fagopyrum esculentum* Moench. & *Fagopyrum tataricum* Gartner.). Curr Adv Buckwheat Res 115: 801–809.

[41] Sircar D, Roychowdhury A, Mitra A (2007) Accumulation of *p*-hydroxybenzoic acid in hairy roots of *Daucus carota* . J Plant Physiol 164: 1358–1366. 17027117 10.1016/j.jplph.2006.08.002

[42] Tsuzuki E, Yamamoto Y (1987) Studies on allelopathy among higher plants V. Isolation and identification of phenolic substances from wild perennial buckwheat (*Fagopyrum cymosum* M.). Bulletin of the Faculty of Agriculture - Miyazaki University. 34: 289–295.

[43] Mandal S, Mitra A (2008) Accumulation of cell wall-bound phenolic metabolites and their upliftment in hairy root cultures of tomato (*Lycopersicon esculentum* Mill.). Biotechnol Lett 30: 1253–1258. 18273552 10.1007/s10529-008-9666-9

[44] Kobayashi S, Nagasawa S, Yamamoto Y, et al (2012) Metabolic profiling and identification of the genetic varieties and agricultural origin of *Cnidium officinale* and *Ligusticum chuanxiong* . J Biosci Bioeng 114: 86–91. 22627050 10.1016/j.jbiosc.2012.02.015

[45] Kim JK, Park SY, Lim SH, et al (2013) Comparative metabolic profiling of pigmented rice (Oryza sativa L.) cultivars reveals primary metabolites are correlated with secondary metabolites. J Cereal Sci 57: 14–20.

