# Correction: Metabolomic Analysis and Phenylpropanoid Biosynthesis in Hairy Root Culture of Tartary Buckwheat Cultivars

**DOI:** 10.1371/annotation/e3bbacf5-42a6-4010-869a-1c999804869f

**Published:** 2013-09-19

**Authors:** Aye Aye Thwe, Jae Kwang Kim, Xiaohua Li, Yeon Bok Kim, Md Romij Uddin, Sun Ju Kim, Tatsuro Suzuki, Nam Il Park, Sang Un Park

The grant and funding organization in the Funding Statement were incorrectly given. The Funding Statement should read: "This work was supported by a grant from the National Institute of Biological Resources (NIBR), funded by the Ministry of Environment (MOE) of the Republic of Korea (NIBR No. 2013-01-052). The funder had taken part in the role of conceive and design the experiment." 

