# Supplementary material for: Metabolomic Analysis and Phenylpropanoid Biosynthesis in Hairy Root Culture of Tartary Buckwheat Cultivars
Source: PLoS One. 2013 Jun 14;8(6):e65349. doi: 10.1371/journal.pone.0065349 (PMC3683005; doi:10.1371/journal.pone.0065349)
Supplement: Table S1 — PCR primer sets for amplification of rol genes. (DOC) [file pone.0065349.s001.doc]

**Table S1.** PCR primer sets for amplification of *rol* genes.

| Primer name Sequences (5′ to 3′) |
| --- |
| *rol* A-F CATGTTTCAGAATGGAATTA  *rol* A-R AGCCACGTGCGTATTAATCC |
| *rol* B-F TCACAATGGATCCCAAATTG  *rol* B-R TTCAAGTCGGCTTTAGGCTT |
| *rol* C-F ATGGCTGAAGACGACCTGTGT  *rol* C-R TTAGCCGATTGCAAACTTGCA |
| *rol* D-F ATGGCCAAACAACTTTGCGA  *rol* D-R TTAATGCCCGTGTTCCATCG |
