# Supplementary material for: Metabolomic Analysis and Phenylpropanoid Biosynthesis in Hairy Root Culture of Tartary Buckwheat Cultivars
Source: PLoS One. 2013 Jun 14;8(6):e65349. doi: 10.1371/journal.pone.0065349 (PMC3683005; doi:10.1371/journal.pone.0065349)
Supplement: Table S2 — Chromatographic and spectrometric data of the 47 identified compounds analyzed by GC-TOFMS. (DOC) [file pone.0065349.s002.doc]

**Table S2.** Chromatographic and spectrometric data of the 47 identified compounds analyzed by GC-TOFMS.

| Compound | RT^a^ | RRT^b^ | Mass fragment^c^ | Quantification ion^d^ |
| --- | --- | --- | --- | --- |
| Pyruvic acid | 4.34 | 0.417 | 115, **174**, 189 | 174 |
| Lactic acid | 4.39 | 0.421 | 117, **147**, 191 | 147 |
| Valine | 5.03 | 0.483 | 130, **146**, 156 | 146 |
| Alanine | 5.09 | 0.488 | **116**, 147, 190 | 116 |
| Glycolic acid | 6.15 | 0.590 | **147**, 177, 205 | 147 |
| Valine | 6.22 | 0.597 | **144**, 156, 218 | 144 |
| Serine | 6.49 | 0.623 | **116**, 132, 147 | 116 |
| Ethanolamine | 6.54 | 0.628 | 100, 147, **174** | 174 |
| Glycerol | 6.55 | 0.629 | 103, 117, **147** | 147 |
| Leucine | 6.56 | 0.630 | 102, 147, **158** | 158 |
| Isoleucine | 7.10 | 0.681 | 147, **158**, 218 | 158 |
| Proline | 7.14 | 0.685 | **142**, 158, 216 | 142 |
| Nicotinic acid | 7.16 | 0.687 | 106, 136, **180** | 180 |
| Glycine | 7.17 | 0.688 | 147, **174**, 248 | 174 |
| Succinic acid | 7.22 | 0.693 | 129, **147**, 247 | 147 |
| Glyceric acid | 7.28 | 0.699 | 133, **147**, 189 | 147 |
| Fumaric acid | 7.42 | 0.712 | 143, 147, **245** | 245 |
| Serine | 7.45 | 0.715 | 147, **204**, 218 | 204 |
| Threonine | 7.59 | 0.728 | 101, 117, **219** | 219 |
| β-Alanine | 8.24 | 0.791 | 147, **174**, 248 | 174 |
| Malic acid | 8.53 | 0.819 | **147**, 233, 245 | 147 |
| Salicylic acid | 9.09 | 0.872 | 135, 149, **267** | 267 |
| Aspartic acid | 9.10 | 0.873 | **100**, 147, 232 | 100 |
| Methionine | 9.13 | 0.876 | 128, 147, **176** | 176 |
| Pyroglutamic acid | 9.16 | 0.879 | 147, **156**, 230 | 156 |
| 4-Aminobutyric acid | 9.17 | 0.880 | 147, **174**, 304 | 174 |
| Threonic acid | 9.26 | 0.889 | **147**, 205, 220 | 147 |
| Arginine | 9.56 | 0.917 | **142**, 147, 162 | 142 |
| Glutamic acid | 9.58 | 0.919 | 128, 156, **246** | 246 |
| Phenylalanine | 10.05 | 0.964 | 100, 192, **218** | 218 |
| *p*-Hydroxybenzoic acid | 10.06 | 0.965 | 193, **223**, 267 | 223 |
| Xylose | 10.11 | 0.970 | **103**, 147, 217 | 103 |
| Asparagine | 10.22 | 0.981 | **116**, 132, 231 | 116 |
| Ribitol | 10.42 | 1.000 | 103, 147, **217** | 217 |
| Vanillic acid | 11.07 | 1.062 | 223, 267, **297** | 297 |
| Glutamine | 11.08 | 1.063 | 147, **156**, 245 | 156 |
| Shikimic acid | 11.19 | 1.074 | 147, **204**, 255 | 204 |
| Citric acid | 11.25 | 1.080 | 147, **273**, 347 | 273 |
| Quinic acid | 11.40 | 1.094 | **147**, 255, 345 | 345 |
| Fructose | 11.46 | 1.100 | **103**, 147, 217 | 103 |
| Fructose | 11.50 | 1.104 | **103**, 147, 217 | 103 |
| Galactose | 11.53 | 1.107 | **147**, 205, 319 | 147 |
| Glucose | 11.57 | 1.110 | **147**, 160, 205 | 147 |
| Syringic acid | 12.03 | 1.154 | 297, 312, **327** | 297 |
| Mannose | 12.04 | 1.155 | **147**, 205, 319 | 147 |
| Inositol | 13.13 | 1.260 | 147, 217, **305** | 305 |
| Ferulic acid | 13.20 | 1.267 | 308, 323, **338** | 338 |
| Tryptophan | 14.04 | 1.347 | **202**, 219, 348 | 202 |
| Sucrose | 16.09 | 1.544 | 147, **217**, 361 | 217 |
| Trehalose | 16.41 | 1.575 | 147, **191**, 361 | 191 |
| Raffinose | 19.45 | 1.867 | 204, **217**, 361 | 217 |

^a^Retention time (min). ^b^Relative retention time (retention time of analyte/retention time of ribitol). ^c^List of the first three ions with the highest intensities. Ions in boldface indicate the most intense product ion. ^d^Specific mass ion used for quantification.
